# Subchondral Bone Regenerative Effect of Two Different Biomaterials in the Same Patient

**DOI:** 10.1155/2013/850502

**Published:** 2013-07-07

**Authors:** Marco Cavallo, Roberto Buda, Francesca Vannini, Francesco Castagnini, Alberto Ruffilli, Sandro Giannini

**Affiliations:** ^1^I Clinic, Rizzoli Orthopaedic Institute, Bologna University, Via Giulio Cesare Pupilli 1, 40136 Bologna, Italy; ^2^Orthopaedics and Traumatology, I Clinic, Rizzoli Orthopaedic Institute, Bologna University, Via Giulio Cesare Pupilli 1, 40136 Bologna, Italy

## Abstract

This case report aims at highlighting the different effects on subchondral bone regeneration of two different biomaterials in the same patient, in addition to bone marrow derived cell transplantation (BMDCT) in ankle. A 15-year-old boy underwent a first BMDCT on a hyaluronate membrane to treat a deep osteochondral lesion (8 mm). The procedure failed: subchondral bone was still present at MRI. Two years after the first operation, the same procedure was performed on a collagen membrane with DBM filling the defect. After one year, AOFAS score was 100 points, and MRI showed a complete filling of the defect. The T2 mapping MRI after one year showed chondral tissue with values in the range of hyaline cartilage. In this case, DBM and the collagen membrane were demonstrated to be good biomaterials to restore subchondral bone: this is a critical step towards the regeneration of a healthy hyaline cartilage.

## 1. Introduction

Osteochondral lesions of the talus (OLT) are defects of the joint surface affecting the cartilage and the subchondral bone [1], mainly due to traumatic etiology. Since the healing abilities of the chondral tissue are limited, the OLT may evolve to osteoarthritis in 17% to 50% of the cases, a not desirable consequence especially in young patients [2, 3]. Many treatments have been proposed in order to solve symptomatology and restore bone and cartilage but most of all have showed remarkable limits, mostly related to technical demanding procedures, iatrogenic morbidity, and generation of nonadequate fibrocartilage [4]. Regenerative techniques, like autologous chondrocyte implantation (ACI) and bone marrow derived cell transplantation (BMDCT), have been developed in order to heal the lesions through hyaline cartilage [4, 5]. The BMDCT is a regenerative technique which has been providing good outcomes without the need for two surgical steps and high costs like ACI procedure. Specifically, according to Giannini's classification [5], lesions deeper than 5 mm represent a critical point for regenerative techniques. They often require a bone graft or some additional was procedures to achieve a good quality subchondral bone, the first step towards the complete healing of the defects [5]. Among these procedures, the demineralized bone matrix (DBM) addition was demonstrated to be osteoinductive [6], while a collagen membrane has been shown to regenerate cartilage and bone [7].

The aim of this case report is to highlight the role of DBM and collagen membrane in bone regeneration after failed osteochondral regenerative procedure.

## 2. Materials and Methods

**Figure 1:**
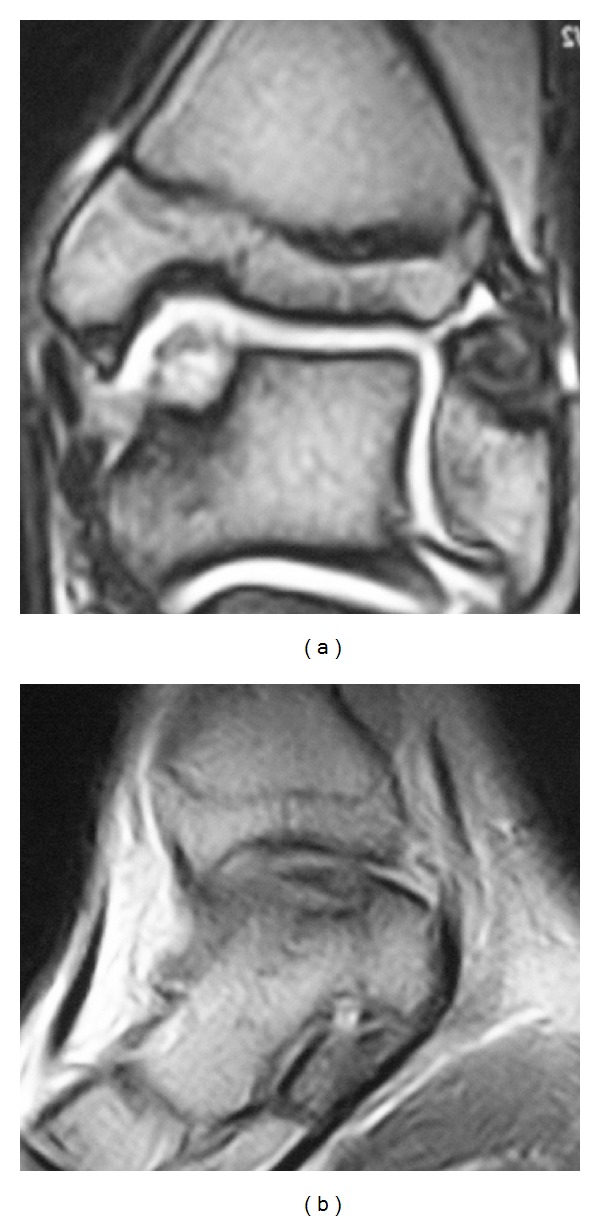
Preoperative coronal (a) and sagittal (b) MRI views before the first operation, showing a IIA type lesion, according to Giannini's classification.

A 15-year-old male arrived at physician attention in 2008. He complained about a persistent ankle pain which had been limiting his sport and daily activities for months. The patient complained of pain on the joint palpation, and the flexion-extension range of motion (ROM) was 20° degree. Pain affected also the stance phase, resulting in an abnormal gait. The AOFAS score [4] was 64 points. An anteroposterior X-ray of the ankle showed an area of rarefaction on the medial area of the talus. MRI confirmed the diagnosis of OLT of the medial talar dome. The lesion size was (16 × 11 mm), and the depth was 8 mm (Figure 1). Due to the dimensions of the lesion and the long lasting severe symptoms, the patient was advised to undergo surgery. 

The day before surgery, through Vivostat system (Vivolution A/S, 3460 Birkerod, Denmark), a sample of 120 mL was taken from the patient's venous blood and then processed as far as 6 mL of platelet rich fibrin (PRF) was obtained. PRF was frozen at −35°C. 

The next day, in a sterile regimen, patient under spinal anesthesia was positioned in prone decubitus. The bone marrow cells aspiration was performed through a marrow needle (size 11 G 9, 100 mm), inserted into the spongy bone of the posterior iliac crest. A total of 60 mL were collected by multiple aspirations of 5 mL of bone marrow each performing withdrawal and rotations to maximize the harvesting and reduce the blood dilution. The aspirated bone marrow was then concentrated through the machine Smart PReP1 (Harvest Technologies Corp., Plymouth, MA, USA). and the dedicated kit BMAC1 (Harvest Technologies Corp.), achieving a 6 mL of bone marrow rich in nucleated cells like stem cells, monocytes, lymphocytes, and bone marrow resident cells and poor in red cells. With patient in supine position, the arthroscopy was performed through two standard approaches, anteromedial and anterolateral. The joint appeared surrounded by fibrous impingement which was removed along with the necrotic subchondral bone and the damaged cartilage. At the same time, 2 mL of the bone marrow concentrate was loaded on a hydrophilic hyaluronate membrane. The biomaterial was sized and shaped following the dimensions of the lesion, previously measured through a millimetered probe. Through a cannula inserted into the trocar of the arthroscopic portal, the biomaterial was finally placed on the lesion with the help of a sliding positioner. The biomaterial was regularized using a flat probe, in order to fill the lesion completely. One mL of PRF was added on the top of the biomaterial to provide extra growth factors and to improve the stability of the membrane. Multiple flexion extensions were performed to check the stability of the implant, and then the portals were sutured. Since the day after surgery, the patient was recommended to perform passive flexion extensions. He was dismissed the first day after the operation. After six weeks, the patient was advised to walk with the crutches, allowing no weight bearing. At eight weeks after the operation, a partial load was allowed. A complete weight bearing was finally achieved at ten weeks after surgery. Swimming, cycling, and other low impact sport activities were allowed at four months after the operation. 

**Figure 2:**
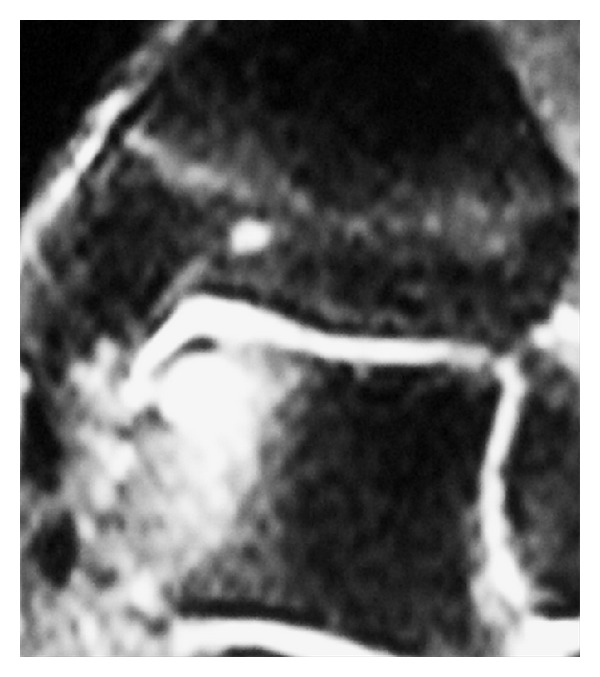
Coronal MRI view 2 years after the first operation. The cartilaginous layer was present, but it was submined by a subchondral cyst.

Nevertheless, the patient did not report the significant improvements expected. Two years after surgery, a moderate pain was still present and no sport activities were tolerated. The ROM was still limited. The AOFAS score was slightly improved from 64 to 74 points. Anteroposterior X-ray showed a medial area of rarefaction, similar to the preoperative projections. MRI scan highlighted a subchondral cyst in the proximity of the medial area which was surgically treated two years before: however, a layer of new cartilage tissue was present (Figure 2). The patient was informed and scheduled for a revision surgery. 

As revision procedure, a bone marrow harvesting was performed from the posterior iliac crest as in the first procedure, and an ankle arthroscopy was performed. A significant fibrous impingement was removed. In the medial area of the talar dome where the lesion was originally located, the formation of new, good-looking cartilage was reported. Nevertheless, the chondral tissue, despite the good integration with the adjacent viable cartilage, looked softened. Under the newly formed layer of cartilage, the condition of the subchondral bone was impaired by the presence of a cyst. The softened cartilage and the subchondral bone were curetted until viable tissues were reached. The dimensions of the debrided lesion were similar to the defect detected two years before, with a lesion depth of 8 mm. Demineralized bone matrix (DBM) was mixed with bone marrow concentrate and put into the lesion to fill the defect; above it, a collagen membrane (Novagenit, Mezzolombardo, Trento, Italy), loaded by bone marrow cells, was positioned. The same arthroscopic devices were used. A layer of PRF was spread on the lesion to provide the growth factors needed. The same rehabilitation program was recommended to the patient. 

**Figure 3:**
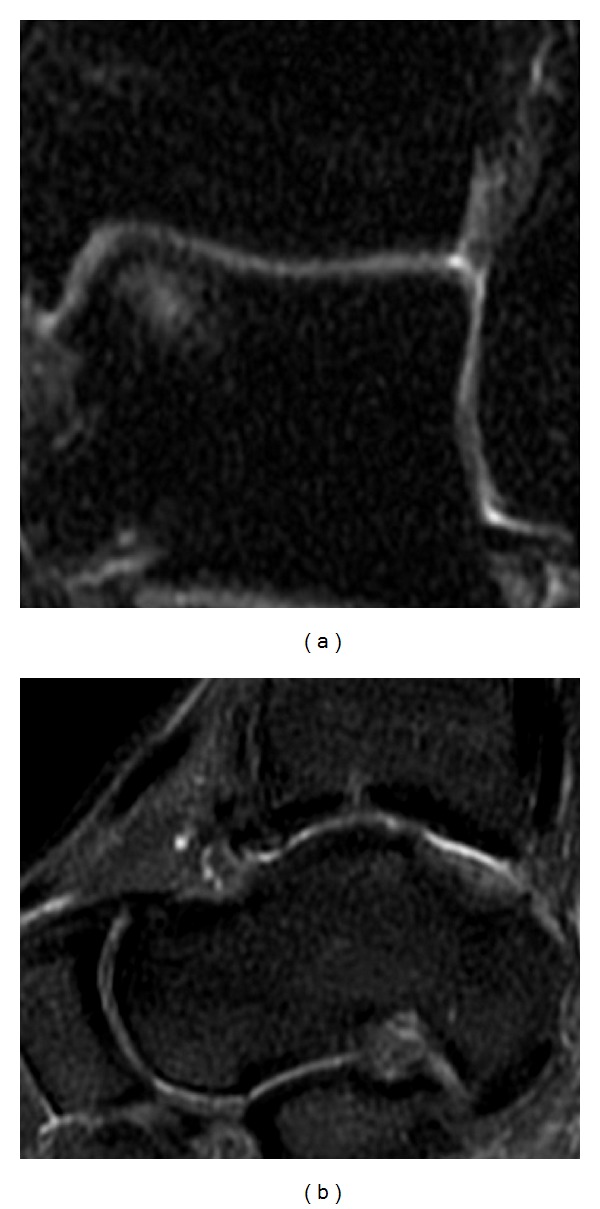
MRI performed 1 year after the second operation, coronal (a) and sagittal (b) views. There are no signs of the previous subchondral cyst. The MOCART scale demonstrates that the defect is completely filled.

**Figure 4:**
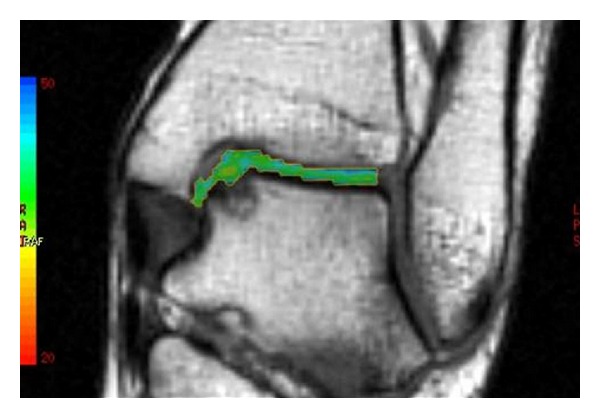
The T2 mapping MRI performed one year after the second treatment shows a 42 msec value, a sign of hyaline cartilage regeneration.

No postoperative complications occurred. Four months after the surgery, the patient could play low impact sports, like swimming and cycling, without any complaints. The ankle pain was extinguished, and the ROM was close to normality. One year after the treatment, the ankle was not painful and the ROM was complete. Due to the good clinical outcomes, the patient was gradually introduced to the high impact sport he played before (soccer, competitive level) one year after the operation. The AOFAS score at one year was 100 points. The MRI performed at one year recorded no signs of the previous cyst. A complete filling of the defect was present, and the chondral tissue was in remodeling phase (Figure 3). The T2 mapping MRI performed at 12 months showed a chondral tissue with a signal of 42 milliseconds, a value that is in the range of normality for hyaline tissue [8] (Figure 4).

## 3. Results and Discussion

The first surgical treatment (bone marrow concentrated cells on a hyaluronate membrane) was inadequate to regenerate a viable subchondral bone, affecting the quality of the cartilage. The second operation, relying on the filling of the bony defect with the osteoinductive abilities of DBM and collagen membrane, succeeded in healing the bony damage, achieving an excellent outcome. Deep lesions require a bone graft and a stimulation of the bone growth in general to succeed in regenerating the osteochondral tissue [5]. The cancellous bone autograft is the gold standard for filling deep subchondral lesions, but it requires a second surgical site for harvesting with a possible local morbidity. Allografts (whole or processed) are in many cases a valuable alternative. DBM is derived from a pulverization of allograft bone [9]: it has been demonstrating good laboratorial and clinical results [10], remarking its osteoinduction [6]. The presence of proteins, collagen, and growth factors permits cellular precursors to be recruited and then differentiated into an osteochondrogenic lineage through the stimulation of the revascularization [11]. In a work by Gao et al. [12], a composite of stem cells and cortical DBM was implanted in rabbits' osteochondral defects of the femoral condyle. The lesions were almost fully filled (95% of the depth) after twelve weeks: the subchondral bone was repaired, and the cartilage appeared to be well integrated. The same experimentation performed with trabecular DBM failed. Moreover, it has been recently found that in experimental settings it is even possible to induce a good activity of chondrocytes if these cells are mixed with DBM, which can offer a good biomechanical stability [13]. DBM has been experimented for the treatment of many kinds of cysts and bony defects, with encouraging results, remarking its osteoinductive properties [14, 15]. The subchondral cysts are close to the cartilage layer, and a proper stimulation for the chondral tissue regeneration may be appropriate. As a matter of fact, in a work by Kolker et al. [16], large and deep OLT were treated with a cancellous bone graft which succeeded in improving the condition of the subchondral bone but regenerated fibrocartilage, affecting the whole outcome. DBM is valuable when a bony regeneration is required, showing no harmful properties for the cartilage. 

Hyaluronate membrane has been intensely adopted to heal osteochondral defects. Many studies highlight its ability to restore chondral tissue inducing formation of hyaline like cartilage [4]. Nevertheless, the bone regeneration driven by hyaluronate composites is a much complex purpose to achieve: only few studies report interesting results, and most of them are about maxillofacial or animal procedures [17, 18]. Collagen membrane is as effective as hyaluronate at stimulating chondral repairing [4, 5]. In this case report, a hyaluronate scaffold did not achieve a good outcome in regenerating bone, while a layer of cartilage was stimulated; nevertheless, collagen membrane with the addition of DBM seemed to provide better results. In a study by Guda et al. [7] with control groups, a collagen membrane was used as a guide for bony regeneration along with hydroxyapatite bone graft: the regenerated bone volume and density were greater and faster. Lin [19] reported good outcomes when expanded mesenchymal stem cells on a collagen membrane were implanted in rabbit's osteochondral defects. Both hyaline cartilage and subchondral bone succeeded in regenerating. A case report reported by Miska et al. [20] dealt with a large sized OLT defect on tibial plafond, treated with autologous matrix-induced chondrogenesis-aided reconstruction. After filling the lesion with a cancellous bone plug from the iliac crest, a collagen matrix I/III was fixed on the defect. After three years, hyaline cartilage over a restored subchondral bone was recorded. All these pieces of evidence stress the role of bone graft, which is a key point to help cartilage regenerate through subchondral bone restoration. Moreover, collagen membrane seems to be very adequate in stimulating the regeneration of cartilage and bone, while evidence of hyaluronate membrane effects is abundant for chondral growth and scarce for bony restoration.

## 4. Conclusions

The aim of this case report is to highlight that the DBM and collagen membrane, due to their osteoinductive properties, may offer a good basis for the filling and the regeneration of the bone and, consequently, may deeply influence the restoration of a viable hyaline cartilage. In fact, the outcome of the first operation, in which no filling of the defect was performed and hyaluronate membrane was the chosen scaffold, was a softened cartilage, a precocious sign of chondral damage. The T2 mapping MRI after the second operation indicated the formation of a cartilage with T2 values suggestive of hyaline tissue, showing that DBM and collagen membrane could provide the regeneration of a viable subchondral bone and a cartilage layer.
